# Entanglement classification with matrix product states

**DOI:** 10.1038/srep30188

**Published:** 2016-07-26

**Authors:** M. Sanz, I. L. Egusquiza, R. Di Candia, H. Saberi, L. Lamata, E. Solano

**Affiliations:** 1 Department of Physical Chemistry, University of the Basque Country UPV/EHU, Apartado 644, 48080 Bilbao, Spain; 2 Department of Theoretical Physics and History of Science, University of the Basque Country UPV/EHU, Apartado 644, 48080 Bilbao, Spain; 3 Department of Optics, Faculty of Science, Palacký University, 17. listopadu 12, 77146 Olomouc, Czech Republic; 4 Department of Physics and Center for Optoelectronics and Photonics Paderborn (CeOPP), University of Paderborn, Warburger Straβe 100, 33098 Paderborn, Germany; 5 IKERBASQUE, Basque Foundation for Science, Maria Diaz de Haro 3, 48013 Bilbao, Spain

## Abstract

We propose an entanglement classification for symmetric quantum states based on their diagonal matrix-product-state (MPS) representation. The proposed classification, which preserves the stochastic local operation assisted with classical communication (SLOCC) criterion, relates entanglement families to the interaction length of Hamiltonians. In this manner, we establish a connection between entanglement classification and condensed matter models from a quantum information perspective. Moreover, we introduce a scalable nesting property for the proposed entanglement classification, in which the families for *N* parties carry over to the *N* + 1 case. Finally, using techniques from algebraic geometry, we prove that the minimal nontrivial interaction length *n* for any symmetric state is bounded by 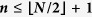.

Entanglement is widely considered the cornerstone of quantum information and an essential resource for relevant quantum effects, such as quantum teleportation^1^,^2^,^3^,^4^, quantum cryptography^5^,^6^, or the speed-up of quantum computing^7^, as in Shor’s algorithm^8^. Moreover, entanglement is recognized as useful for understanding properties of condensed matter models, such as quantum phases^9^ and topological orders^10^, among others. Entanglement based properties are usually challenging to study both experimentally and theoretically, due to the exponential growth of the associated quantum degrees of freedom. Experimentally, due to the exponentially large amount of degrees of freedom typically involved, the detection and the quantification of the entanglement are difficult to achieve. Theoretically, quantities describing the entanglement are generally a complicated function of the quantum state, and they are normally arduous to analyse. To overcome these obstacles, advanced quantum information techniques have been successfully applied to answer condensed matter questions^11^,^12^,^13^, shedding a distinct light on the problem. This novel approach may bring about exciting results in many-body physics, and result in a new revolution in physics, where quantum information and matter phenomena can be formally unified^14^.

An important question in quantum information is the classification of entanglement by means of some mathematical or physical equivalence. Classifying entanglement should help in recognizing similarity between different entangled states, and it may be useful to boost the practicabilities of quantum information protocols. A first result is that quantum states connected by SLOCC operations, which perform probabilistically the same quantum tasks, can be collected into entanglement classes, called SLOCC classes, but also known as SLOCC criterion^15^. Nevertheless, there is an infinite number of SLOCC classes for four or more parties that may be gathered, in turn, into a finite number of entanglement families^16^,^17^,^18^,^19^,^20^,^21^. Unfortunately, the community has not been able to relate all classes and families to specific properties or quantum information tasks, although a few of them have certainly raised experimental interest^22^,^23^,^24^,^25^. It is noteworthy to mention that, up to now, no general characterization nor classification of entanglement exist for many-body systems.

In this Article, we present an entanglement classification for quantum states induced by their MPS structure, which preserves the SLOCC criterion and is exemplified for the symmetric subspace. The proposed classification is based on the local properties of the multipartite quantum state. In this sense, it relates entanglement families to the interaction length of Hamiltonians, establishing a connection between entanglement classification and condensed matter. Our proposal is twofold beneficial: on the one hand, it does not result in an infinite number of entanglement classes, considerably simplifying their study; on the other hand, it provides a direct physical insight to the nonlocality of entanglement classes, given by the interaction length of their parent Hamiltonians. Additionally, we introduce a scalable nesting property in which the families for *N* parties carry over to the *N* + 1 case.

We focus on the classification of the SLOCC classes corresponding to symmetric states, which are invariant under any permutation of the parties^26^, i.e. *F*|*ψ*〉 = |*ψ*〉 where *F* is an exchange operator. This is an interesting subspace, since its dimension grows linearly with the number of parties but, at the same time, it contains many physically relevant states.

A pure state |Ψ〉 is called *entangled* if it is not *separable*^27^, i.e. if it cannot be written as a tensor product 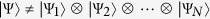. This definition is indirect and this may be the reason that its quantification for more than two parties is still unsettled. The idea of separability emerges when one tries to identify which states can be generated from other states, defined locally in each subsystem, by using local operations and classical communication among parties. Therefore, these local operations provide a natural criterion to collect quantum states in *entanglement classes* with the same type of entanglement^15^. More specifically, two states belong to the same class if they can be transformed into each other with non-zero probability via SLOCC operations.

The splitting of the Hilbert space into the SLOCC classes is fully understood for bipartite and tripartite qubit systems, even in the nonsymmetric case^15^. There is only one entanglement class for two qubits: the one containing the Einstein-Podolski-Rosen (EPR) state 
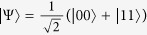
, and two symmetric classes for three qubits: one represented by the Greenberger–Horne–Zeilinger (GHZ) state 
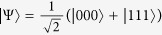
, and the second one referenced to the *W* state 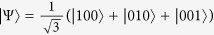. However, for four or more qubits, the number of SLOCC classes is infinite, and their parametrization grows exponentially with the number of parties, while lacking robustness against experimental errors^15^. In this sense, SLOCC classification makes the association of classes to specific physical properties difficult. This explains, so far, the lack of experimental interest in producing states beyond the well-known GHZ or *W*, among few others.

Due to the natural interest of the SLOCC criterion, it is customary to collect these infinite SLOCC classes into a finite number of larger families based on specific mathematical properties^16^,^17^,^18^,^20^ or operational approaches^21^,^28^ (see Fig. 1a). However, up to now, all classifications have failed to associate groups of classes or families to specific quantum information tasks. Since there are infinite SLOCC classes for four or more parties, they can be partitioned into families in an infinite number of ways, and we would not expect all to be relevant. To overcome this conundrum, we propose the following criteria that an SLOCC classification into families should fulfill: (1) Every SLOCC class must belong to only one family (classes should not cross any border separating families), (2) separable states must be in one family and this family should contain only separable states, (3) SLOCC classes belonging to the same family must show common physical and/or mathematical properties, and (4) the classification into families must be efficient in the sense that (a) the number of families must grow “slowly” with the number of qubits, and (b) the efforts for classifying *N* qubits should be useful for classifying *N* + 1 (nesting).

In the last decades, matrix product states (MPS) and tensor networks have emerged as a powerful tool to tackle complex problems in many-body systems^29^,^30^,^31^,^32^. In this sense, any quantum state 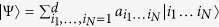 can be rewritten in a local manner as 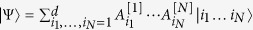, where 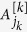 are matrices containing all local information related to site *k*. This language is convenient to describe ground states of local Hamiltonians^30^,^31^,^33^, sequential processes^34^,^35^,^36^, and systems fulfilling an area law^37^. In the following, we use the MPS formalism to define an entanglement classification of quantum states into families of SLOCC classes, based on the local dimension of the matrices describing the states, called *bond dimension* (see Fig. 1b). Furthermore, we prove that this MPS classification is directly related to the interaction length of the corresponding parent Hamiltonian^31^. We apply these novel concepts to the case of the symmetric subspace, although they could be extended to more restricted or more general sets of states. We start by highlighting that any symmetric state admits MPS representations with site-independent diagonal matrices. If we further request a minimal bond dimension, we must find the optimal way to represent any symmetric state as 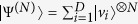. The dimension of the matrix parallels the number of the Schmidt coefficients, which has been proposed to quantify entanglement^38^. Hereafter, when we refer to the bond dimension of a symmetric state, we mean the *minimal* bond dimension associated with its diagonal representation.

Crucially, SLOCC transformations preserve the minimal bond dimension (see Supplementary Information). Indeed, if 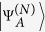 and 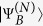 are two quantum states with bond dimensions *D*_*A*_ and *D*_*B*_ respectively, and 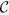 a class, then


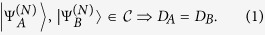


This implies that all states of the same SLOCC class may be represented with the same minimal matrix dimension, which is a SLOCC invariant. In this way, we can define a family of SLOCC classes by means of the following equivalence relation:

**Definition** (Diagonal MPS entanglement classification). *Let S*_*A*_ *and S*_*B*_ *be SLOCC classes, and D*_*A*_*, D*_*B*_ *the minimal bond dimension of their respective states in its diagonal MPS representation. We say that* 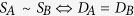
*, and we call entanglement families the resulting equivalence classes*.

Notice that the class of separable states can be optimally represented with matrices with bond dimension *D* = 1, and, indeed, it coincides with the family *D* = 1. Therefore, the diagonal MPS (DMPS) classification, proposed here for the symmetric subspace, fulfills the first two aforementioned conditions. Moreover, in this DMPS classification any symmetric state of *N* qubits can be written with at most bond dimension *N*, so the number of families grows linearly with the number of parties involved. In this sense, the DMPS classification also satisfies a recently proposed tractability criteria^20^.

The explicit translational invariance of the MPS formulation leads the DMPS classification to fulfill the aforementioned criterion (4b). Let {*A*_*i*_}_*i*_ define an *N*-partite symmetric state 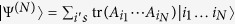. Then, the state 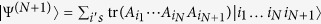, which lives in a different Hilbert space, namely that of *N* + 1 parties, does show exactly the same local properties as |Ψ^(*N*)^〉. As the DMPS classification respects these local properties, and not just for a given state but for the whole SLOCC class, an intriguing *nesting property* of the families for different number of parties emerges:

**Theorem** (Nesting) *Let us consider an N-particle symmetric state of qubits* 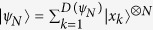 *with optimal bond dimension D*(*ψ*_*N*_)*, such that* 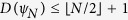
*. Then, the state* 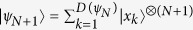 *has optimal bond dimension* 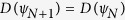.

See Supplementary Information for the proof. This theorem shows that, from the perspective of the local properties, the only purely *N*-partite states are the ones whose optimal bond dimension is larger than the maximal bond dimension of any state with *N* − 1 parties. This generates a *matryoushka structure* depicted in Fig. 1b, where, unlike other entanglement classifications, the classification for *N* parties is connected with the classification for *N* + 1, for all *N*. We believe that a further exploitation of this scalable nesting property would be interesting in the many-body case, where the exact number of particles is usually not relevant.

Lastly, in the MPS formalism, the role of parent Hamiltonians for a given state comes to the fore. Namely, for any given state, one can construct a local Hamiltonian which includes it in its ground eigenspace. The MPS representation informs us about the features and interaction length of this construction. For instance, it controls whether it is the only ground state or there is a spontaneous symmetry breaking^29^, or the inheritance of local and global symmetries^30^,^31^. This constructive method works as follows: Let |Ψ^(*N*)^〉 be a symmetric pure state of *N* parties with an MPS representation with bond dimension *D*. The reduced density matrices for *n* ≤ *N* are defined by 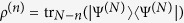, i.e. by tracing out *N* − *n* parties. By construction, rank(*ρ*^(*n*)^) ≤ *D*, so when *n* > log_2_*D*, *ρ*^(*n*)^ has a kernel. Let 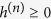 be the projector onto this kernel and 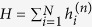 the total Hamiltonian. Thus, *H* is a positive operator and 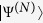 is in its ground manifold since *H*|Ψ^(*N*)^〉 = 0. Additionally, the interaction length of *H* is *n*. In order to apply this construction to the DMPS classification, notice that all reduced density matrices for more than one party have nontrivial kernel when acting on the full *n*-qubit Hilbert space, since their support is restricted to symmetric states. We must then consider the relevant kernel in the corresponding symmetric subspace of dimension *n* + 1. Clearly, if the bond dimension is *D*, the rank of *ρ*^(*n*)^ is at most *D* in the symmetric space as well. Thus, we have ensured the existence of a relevant parent Hamiltonian with interaction length *n*, if *n* ≥ *D*. However, techniques from algebraic geometry allow us to prove that the minimal nontrivial interaction length for any symmetric state is bounded as 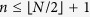 (see Supplementary Information). We shall see from the examples below, indeed, that interaction lengths shorter than *D* do arise. Notice that in our construction we propose families of parent Hamiltonians, for all of which all the states in an entanglement family are ground states. In particular, the ground manifold is always degenerate in our construction. To put this statement in context, bear in mind that the concept of parent Hamiltonian of a state is a general one: a local Hamiltonian which includes the state in its ground manifold. A particularly well established technique for constructing parent Hamiltonians for states or families of states is given by the MPS construction. In that case, the uniqueness of the ground state of this MPS Hamiltonian will depend on certain properties of the corresponding matrix. In particular on the property (or otherwise) of injectivity, such that a lower bound for the interaction length is given by a polynomial in *D* and *d* (see ref. 39). A particular example of this for the GHZ state may be found in ref. 40

(*i*) *GHZ states*.*—* The GHZ states may be represented, independently of *N*, by the matrices 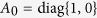 and 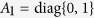. The corresponding parent Hamiltonian family reads


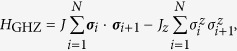


with the conditions *J*_*z*_ > 0 and *J*_*z*_ > 2*J*.

(*ii*) *W states*.*—* The *W*_*N*_ states generalize to *N* parties the structure of the three-partite *W* state, and they correspond to Dicke states with just one excitation. They can be represented with bond dimension *D*(*W*_*N*_) = *N* by the following sequence of diagonal matrices 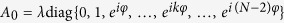, with 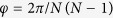, and 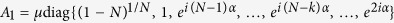, where *α* = 2*π*/*N*. Then, we propose the parent Hamiltonian family


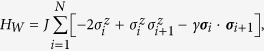


where both *J* and *γ* need to be positive. Notice that, even though the *W* state requires a bond dimension *N* to be represented with diagonal matrices, we can find a Hamiltonian with interaction length 2 which has this state as a ground state.

(*iii*) *X*_*N*_ *states*.*—* Let us consider the family for *N* ≥ 4 given by


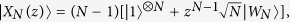


up to normalization. The DMPS representation of this state is given by the (*N* − 1)-dimensional matrices 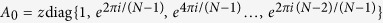 and 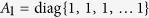. In this case, the relevant interaction length is seen to be 3. In fact, the parent Hamiltonian family reads


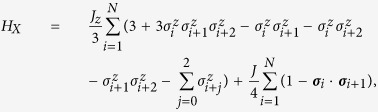


for any *J* > 0 and *J*_*z*_ > 0.

In conclusion, the proposed MPS classification fulfills all the aforementioned criteria for a versatile grouping of SLOCC classes, while maintaining a linear growth in the number of families with respect to the number of parties. At the same time, the unveiled nesting property allows us to use, in a scalable manner, the effort invested in the MPS classification for *N* parties in the subsequent *N* + 1 case. Additionally, we have provided a physical meaning for the proposed MPS classes by connecting them to paradigmatic properties in condensed matter. This missing link in the theory of entanglement classification has been exemplified for the case of the symmetric subspace, where the DMPS representation was used. It is noteworthy to mention that the provided DMPS classification can be formally extended from the symmetric subspace to more general sets of quantum states. We believe that MPS-based entanglement classifications will be able to connect mathematical aspects already known in quantum information theory with relevant physical features in many-body systems of experimental interest, such as quantum state preparation or the emergence of fractional magnetizations.

## Additional Information

**How to cite this article**: Sanz, M. *et al*. Entanglement classification with matrix product states. *Sci. Rep.* **6**, 30188; doi: 10.1038/srep30188 (2016).

## Supplementary Material

Supplementary Information

## Figures and Tables

**Figure 1:**
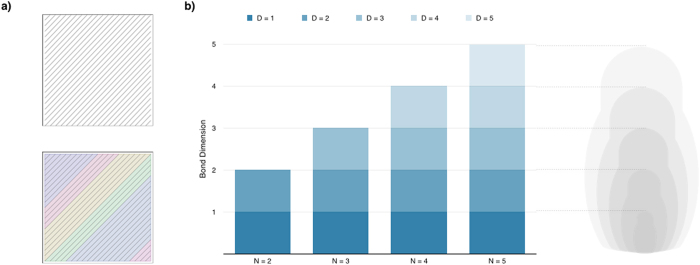
(**a**) The SLOCC criterion divides the Hilbert space (the square) in such a way that every quantum state is in a well defined class (the lines). For four or more parties, the number of these SLOCC classes is infinite. However, they may be gathered into families (the colored areas) under certain rules, ideally with more physical associations than mathematical ones. Here, the condition is given by the minimal bond dimension of the matrix-product-state representation of quantum states, relating the MPS classes to the interaction length of parent Hamiltonians. (**b**) The proposed MPS classification enjoys a scalable nesting property in which the classes of an *N*-partite family can be mapped onto the classes of the corresponding (*N* + 1) case, generating a *matryoushka structure*. A detailed example is given for the symmetric subspace of arbitrary number of parties.
